# Simplified primer design for PCR-based gene targeting and microarray primer database: two web tools for fission yeast

**DOI:** 10.1002/yea.1422

**Published:** 2006-10-15

**Authors:** Christopher J Penkett, Zoë E Birtle, Jürg Bähler

**Affiliations:** Cancer Research UK Fission Yeast Functional Genomics Group, Wellcome Trust Sanger InstituteCambridge CB10 1HH, UK

**Keywords:** primer sequence, *Sz. pombe*, gene deletion, gene expression, gene tagging, microarray, quantitative PCR

## Abstract

PCR-based gene targeting is a popular method for manipulating yeast genes in their normal chromosomal locations. The manual design of primers, however, can be cumbersome and error-prone. We have developed a straightforward web-based tool that applies user-specified inputs to automate and simplify the task of primer selection for deletion, tagging and/or regulated expression of genes in *Schizosaccharomyces pombe*. This tool, named PPPP (for Pombe PCR Primer Programs), is available at http://www.sanger.ac.uk/PostGenomics/S_pombe/software/. We also present a searchable Microarray Primer Database to retrieve the sequences and accompanying information for primers and PCR products used to build our in-house *Sz. pombe* microarrays. This database contains information on both coding and intergenic regions to provide context for the microarray data, and it should be useful also for other applications, such as quantitative PCR. The database can be accessed at http://www.sanger.ac.uk/PostGenomics/S_pombe/microarray/. Copyright © 2006 John Wiley & Sons, Ltd.

## Introduction

A major strength of yeast genetics is the ease with which specific genes can be manipulated within the genome, which greatly facilitates functional analyses. Gene targeting takes advantage of homologous recombination between transformed DNA fragments that terminate in short stretches of target sequence and the corresponding genomic sites (Rothstein, 1983; Grimm and Kohli, 1988). The most straightforward approach for gene targeting is to design long primers that include the target sequences, and then carry out PCR with these primers to generate the DNA fragment for transformation (Baudin *et al.*, 1993; Wach *et al.*, 1997). Modular and versatile constructs are available for gene deletion, gene tagging and regulated gene expression in fission yeast (Bähler *et al.*, 1998; Tasto *et al.*, 2001; Sato *et al.*, 2005; Hentges *et al.*, 2005; Van Driessche *et al.*, 2005). These constructs contain different types of markers, tags and promoters; they can be amplified by PCR using a limited number of primers, thus reducing costs and increasing flexibility for analysing gene function. The design of PCR primers involves the selection of appropriate target sequences relative to the gene of interest (typically ∼80 bp) combined with the sequences to amplify the constructs (∼20 bp). Manual primer selection is therefore quite complicated, and any mistakes lead to time loss at best and misleading results at worst. To ease this laborious task, we have developed a web-based tool (Pombe PCR Primer Programs; PPPP) that automatically suggests primer sequences for deletion, tagging and regulatable expression of genes, based on the gene name, length of target sequence and plasmid information that the user specifies. In addition, the tool can design short primers up- or downstream of the target sequences to screen by PCR for correct homologous integration of the transformed fragments. Our group and others now routinely use this tool to facilitate the reliable design of primers for gene targeting.

We also describe a searchable Microarray Primer Database that contains information on primers and PCR products used for our in-house *Sz. pombe* microarray platform. The intragenic (coding) regions have been used on microarrays for expression profiling studies (Mata *et al.*, 2002; Smith *et al.*, 2002; Chen *et al.*, 2003; Mata and Bähler, 2003; Rodríguez-Gabriel *et al.*, 2003; Gatti *et al.*, 2004; Rustici *et al.*, 2004; Sanders *et al.*, 2004; Watson *et al.*, 2004; Hansen *et al.*, 2005; Harrison *et al.*, 2005; Jenkins *et al.*, 2005; Lee *et al.*, 2005; Mandell *et al.*, 2005; Bachand *et al.*, 2006; Martín *et al.*, 2006; Rodríguez-Gabriel *et al.*, 2006; Sharma *et al.*, 2006; Mata and Bähler, 2006). We have also started to use microarrays covering all intergenic regions for complementary genome-wide studies (e.g. Heichinger *et al.*, 2006). Comprehensive data on intra- and intergenic regions are provided in the Microarray Primer Database to look up particular features of regions of interest represented in the microarray data. Given that primers were selected to cover sequences without cross-hybridization to other genomic sequences and to be located within exon sequences if used for expression profiling (Lyne *et al.*, 2003), the database could also be used for other applications, e.g. to select primers for quantitative PCR.

## Implementation

**Figure 1:**
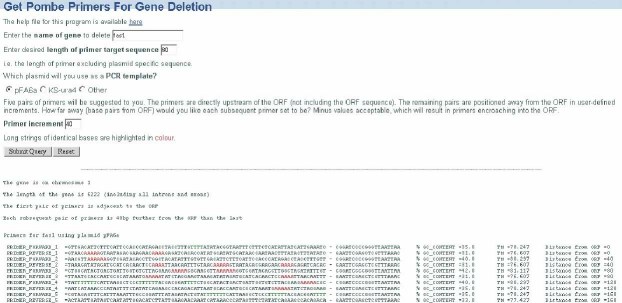
Screenshot of PPPP output page, using the gene deletion mode with the *fas1* gene as an example

**Figure 2:**
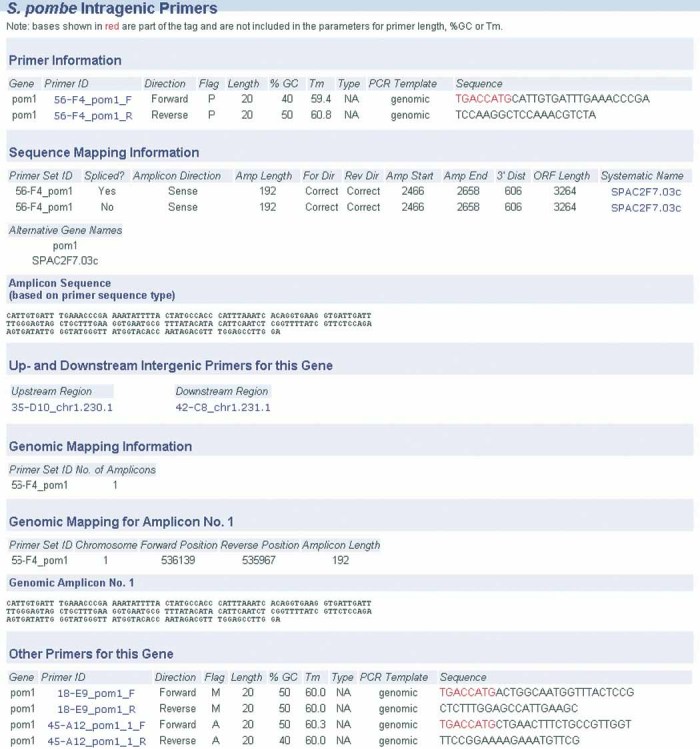
Screenshot of output page from Microarray Primer Database. Full page for intragenic search, using the *pom1* gene as an example. The list for other primers at the bottom is only partially shown

**Figure 3:**
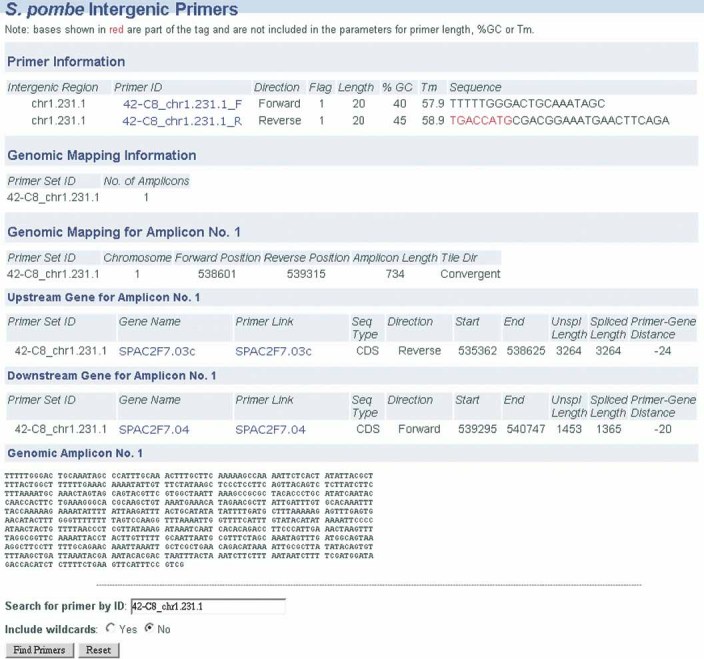
Screenshot of output page from Microarray Primer Database. Full page for intergenic region between *pom1* and *pmc2* genes. This page has been accessed by clicking on intergenic primer for ‘Downstream Region’ in the page shown in Figure 2

Both PPPP and the Microarray Primer Database are written in Perl. We have designed web interfaces for both web tools using the CGI module of Perl, hosted on an Apache server. Genes can be searched using any of the gene names or systematic identifiers available in GeneDB (http://www.genedb.org/genedb/pombe/; Hertz- Fowler *et al.*, 2004). The outputs of both tools are provided in tabulated HTML format (Figures 1, 2 and 3).

PPPP stores the gene locations and directions, along with gene synonyms, in a Perl disk-based hash (DBM or DataBase Module) file. This file is pre-calculated from data in *Sz. pombe* GeneDB to speed up the program for primer design without the overhead of creating a relational database. When the program is run, the gene locations are used to extract sequences from the *Sz. pombe* genome (Wood *et al.*, 2002), from which the primers are designed.

The Microarray Primer Database is implemented as a MySQL relational database running on a UNIX server. The database was created using a Perl-based pipeline with the sequences of the primers used for the intra- and intergenic arrays. Primers are mapped to the genome and, for the intragenic primers, have been checked to map to the correct gene. A set of parameters for each primer is then calculated, including length, GC percentage and melting temperature (*T*_m_) for short primers (Breslauer *et al.*, 1986). Finally, the amplicon sequences are extracted from genomic DNA (or from cDNA sequence if a primer pair flanks intron sequence). Once all of this information has been collected, it is imported into the MySQL database using the Perl DBI module.

The intragenic primers have been designed as described by Lyne *et al.* (2003). The intergenic primers have been designed using Perl scripts that check EMBL sequence files for regions between any open reading frames (ORFs) and known non-coding RNA genes. Long intergenic regions are divided into sub-sections to increase microarray resolution, as follows. For regions between divergently expressed genes, one section is used for regions up to 1000 bp, whilst regions between 1000 and 4000 bp are divided into two sections. For regions between tandem or convergent genes, one section is used for regions up to 2000 bp, whilst regions between 2000 and 4000 bp are divided into two sections. In all cases, longer regions are divided such that no sub-section is longer than ∼2000 bp. The Perl scripts for the design of intra- and intergenic primers can be found under ‘Other scripts’ on our software page (http://www.sanger.ac.uk/PostGenomics/S_pombe/software/); this page also includes PPPP (this report), YOGY (for integrated orthology and Gene Ontology analyses; Penkett *et al.*, 2006) and a script for initial microarray data processing (Lyne *et al.*, 2003).

## Web tool description

### Pombe PCR primer programs

The PPPP entry page (http://www.sanger.ac.uk/PostGenomics/S_pombe/software/) provides four separate links to obtain primers for: (a) gene deletion; (b) C-terminal tagging; (c) controlling genes by *nmt1* promoter and/or N-terminal tagging; or (d) checking for homologous integration by PCR. For the first three cases, the following information is required as input:

1. Name of gene to be manipulated.
2. Length of genomic target sequence (excludes the plasmid-specific sequence; default is 80).
3. Plasmid to be used as PCR template (refers to plasmids published by Bähler *et al.*, 1998, although primers will work for any compatible plasmids; ‘other’ means no plasmid-specific sequence will be added; for N-terminal tagging, a tag must be specified).
4. Increment length (see below; default is 40).

Forward and reverse primer sequences are given in the output page (Figure 1). The first set of primers is directly adjacent to the ORF, while the four subsequent primer sets are further away from the ORF at a user-specified distance, depending on the value of the increment used. For example, if the primer increment is set to 40, the second set of primers begins 40 bp before and after the ORF, the third set is placed at 80 bp, etc. A negative increment value results in primers that impinge into the ORF. This allows for a choice of primers with varying positions and base composition. Long strings of identical bases (four nucleotides or more) are highlighted in colour. Additional information is provided on the automatically selected primers as follows:

1. GC content calculated as percentage of total nucleotide content.
2. Melting temperature (*T*_m_), as defined for long primers by Sambrook *et al.* (1989).
3. Distance of primer from the ORF.

The gene deletion program gives a selection of both forward and reverse primers. For C-terminal tagging, only one forward primer is provided as this is the only option for tagging of full-length proteins. Similarly, for *nmt1* regulation/N-terminal tagging, only one reverse primer is provided, which allows manipulation of full-length proteins.

PPPP can also design short PCR primers based on user-defined inputs to check for correct homologous integration of the gene targeting fragments. For this, primers of suitable melting temperature are selected within 1 kb either up- or downstream of the target gene (pointing towards the gene in each case), such that they do not overlap with the target sequence but are close to it. Using these primers in combination with appropriate universal primers within the targeting fragment will result in diagnostic PCR products if the fragment is integrated at the correct genomic location.

### Microarray primer database

The entry page of this tool contains input fields for both intra- and intergenic primers (http://www.sanger.ac.uk/PostGenomics/S_pombe/micro-array/). Intragenic primers can be accessed by searching with any valid *Sz. pombe* GeneDB name including the systematic identifier. If the search identifies more than one set of primers, a list of all primers is provided with basic information on each primer. The database can also be searched using incomplete names with a wild-card option, and again a list of primers is presented if multiple genes represent the incomplete name. The full information pages for the primer sets in these lists can then be obtained by clicking on the hyperlinks associated with the primer names. The search leads directly to the full page if only one primer pair is found. A list of synonyms and systematic identifiers for the gene associated with the primer set is provided on the full page to ensure that the correct gene is selected. Intergenic primers are named by the number of the corresponding intergenic region along the chromosome. Hence, it is easier to access a particular intergenic region by searching for the gene name up- or downstream of the region of interest using the intragenic search; in the intragenic primer output page, the corresponding intergenic regions can then be accessed via hyperlinks to the up- or downstream intergenic region for this gene (see below).

The full page for the intragenic search begins with a table of basic primer information (Figure 2). The primer names include 96-well plate numbers and plate positions used for microarray printing. In addition, the following basic information is included:

1. Primer direction relative to the gene (forward and reverse primers are given on separate lines).
2. Flag for information on PCR products (P, present; M, multiple bands; A, absent). A few primers have been designed with an old version of the genome and were either incorrect or better primers have been designed; in these cases, the old primers are marked as A.
3. Primer length (bp).
4. GC percentage.
5. Melting temperature (*T*_m_).
6. Primer type: indicates if sequence flanked by primers includes introns (cDNA required for PCR template) or not (NA: genomic DNA can be used for PCR template).
7. PCR template used to amplify the primers for the arrays.
8. Primer sequence.

Some primers contain an additional universal sequence, highlighted in red, which does not correspond to *Sz. pombe* genomic sequence. This sequence has been used for second-round PCR amplification with an amino-linked universal primer (Lyne *et al.*, 2003). The universal sequences are not included in the information for primer length, GC content or *T*_m_.

The first table is followed by a table of sequence mapping information for the gene associated with the primer pair (Figure 2). Two lines of data are in this table, one for the spliced and one for the unspliced version of the gene. The mapping data in both lines are identical if both primers are located within the same exon. The following information is provided:

1. Amplicon direction relative to gene (‘sense’: amplicon strand on array measures transcript in direction of gene; ‘anti-sense’: amplicon strand on array measures reverse transcript relative to gene).
2. Amplicon length in bp (PCR product).
3. Direction of forward and reverse primers, indicating whether primers are correctly orientated relative to each other (both ‘correct’; a few primers are wrongly orientated, indicated by ‘wrong’; these will all have correct alternative primers).
4. Amplicon start position within gene relative to start codon ATG (where A is 1).
5. Amplicon end position within gene relative to start codon ATG (where A is 1).
6. Distance (bp) of amplicon end to final base of stop codon.
7. ORF length (bp).
8. Systematic name of gene that primers are mapped to with a link to the corresponding page in *Sz. pombe* Gene DB.
9. Table with alternative names for this gene as given in *Sz. pombe* GeneDB.
10. Formatted version of amplicon sequence excluding any introns.

This gene information is followed by genomic context data (Figure 2). The first table has links to the intergenic regions that are up- and downstream of the mapped gene, which is the easiest way to access intergenic primers of interest. Next, there is a table indicating how many amplicons are possible with the primer pair. Normally this will be 1, but sometimes both primers map to multiple neighbouring positions within the genome that could give rise to multiple amplicons by PCR. Amplicons that would be larger than 4000 bp are not considered.

Next, the following information is shown for each possible amplicon:

1. Mapped chromosome.
2. Positions of forward and reverse primers within this chromosome using *Sz. pombe* GeneDB coordinates.
3. Amplicon length (bp).

A formatted version of the sequence is then given for each amplicon, including any potential introns (Figure 2). If there are multiple primer sets for the gene, a last table on the output page provides a summary of information for the other primer sets available. The format of this table is the same as for the initial list page.

The full page for the intergenic search also begins with a table of basic primer information (Figure 3). The next table indicates how many amplicons are possible with this primer pair (again, only for amplicons up to a maximum length of 4000 bp). For each amplicon, a summary of genomic mapping information is then shown. This is identical to the table produced for intragenic primers. In addition, it also includes information on the direction of the two genes that flank the intergenic region: ‘tandem’, both genes are in same direction, thus the intergenic region contains a single promoter; ‘convergent’, the 3′ ends of both genes point towards the intergenic region, which therefore contains no promoter; ‘divergent’, the 5′ ends of both genes point towards the intergenic region, which therefore contains two promoters. This is followed by two tables, the first for the upstream gene and the second for the downstream gene. No genes are shown in the tables in cases where the primers are flanked by > 20 000 bp of intergenic sequence. Both tables for flanking genes contain the following information:

1. Link to the gene in *Sz. pombe* GeneDB.
2. Link to the primers available for this gene.
3. Type of sequence for this gene as specified in *Sz. pombe* GeneDB (e.g. CDS, tRNA, rRNA).
4. Direction of the gene relative to the chromosome as specified by *Sz. pombe* GeneDB.
5. Start and end positions of the gene within the chromosome using *Sz. pombe* GeneDB coordinates.
6. Unspliced length of the ORF (bp).
7. Spliced length of the ORF (bp).
8. Shortest distance of primer from end of the closest ORF (a negative value indicates that this primer overlaps with the ORF).

For each possible intergenic amplicon, a formatted version of the amplicon sequence is then presented at the bottom of the page (Figure 3).

## Conclusions

PPPP makes primer design for a range of PCR-based gene targeting approaches less painful and more reliable, while the Microarray Primer Database provides searchable information on primers and PCR products used to generate our microarray data and can also help for other applications. We regularly take advantage of these web tools and hope that colleagues of the fission yeast community will find these tools similarly useful for their research.
